# EnzyNet: enzyme classification using 3D convolutional neural networks on spatial representation

**DOI:** 10.7717/peerj.4750

**Published:** 2018-05-04

**Authors:** Afshine Amidi, Shervine Amidi, Dimitrios Vlachakis, Vasileios Megalooikonomou, Nikos Paragios, Evangelia I. Zacharaki

**Affiliations:** 1 Massachusetts Institute of Technology, Cambridge, MA, USA; 2 Center for Visual Computing, Department of Applied Mathematics, Ecole Centrale de Paris (CentraleSupélec), Châtenay-Malabry, France; 3 MDAKM Group, Department of Computer Engineering and Informatics, University of Patras, Patras, Greece

**Keywords:** Deep learning, 3D convolutional neural networks, EnzyNet, Enzyme classification

## Abstract

During the past decade, with the significant progress of computational power as well as ever-rising data availability, deep learning techniques became increasingly popular due to their excellent performance on computer vision problems. The size of the Protein Data Bank (PDB) has increased more than 15-fold since 1999, which enabled the expansion of models that aim at predicting enzymatic function via their amino acid composition. Amino acid sequence, however, is less conserved in nature than protein structure and therefore considered a less reliable predictor of protein function. This paper presents EnzyNet, a novel 3D convolutional neural networks classifier that predicts the Enzyme Commission number of enzymes based only on their voxel-based spatial structure. The spatial distribution of biochemical properties was also examined as complementary information. The two-layer architecture was investigated on a large dataset of 63,558 enzymes from the PDB and achieved an accuracy of 78.4% by exploiting only the binary representation of the protein shape. Code and datasets are available at https://github.com/shervinea/enzynet.

## Introduction

The exponential growth of the number of enzymes registered in the Protein Data Bank (PDB) urges a need to propose a fast and reliable procedure to classify every new entry into one of the six standardized enzyme classes denoted by the enzyme classification number (EC): Oxidoreductase (EC1), Transferase (EC2), Hydrolase (EC3), Lyase (EC4), Isomerase (EC5), and Ligase (EC6). Previous work, such as by Kumar & Choudhary (2012), included the use of traditional machine learning techniques requiring a tedious and crucial feature extraction step based on amino acid sequence alignment or using structural descriptors without relying on sequence alignment (Dobson & Doig, 2005). A summary of the main alignment-free methods for EC can be found in Amidi et al. (2016), while a systematic review on the utility and inference of various machine learning techniques for functional characterization is presented in Sharma & Garg (2014) and in Yadav & Tiwari (2015). Artificial neural networks (ANNs) in particular have often been used in the past as predictive tools in enzyme activity and catalytic studies. Baskin, Palyulin & Zefirov (2008) reviews several aspects in building quantitative structure-activity relationship (QSAR) models using ANNs, while Szaleniec (2012) focuses on the application of ANNs in QSAR modeling in enzyme reactivity prediction and relevant methodological issues that arise. In a recent review from Li, Zhang & Liu (2017), the effectiveness of ANNs for catalysis prediction and design of new catalysts is discussed from the perspective of both experiment and theory and common challenges are presented.

A limitation in standard machine learning approaches is that the features have to be predefined, the appropriate choice of features affects the prediction accuracy and there is limited flexibility for model changes or updates (all preprocessing steps have to be repeated). These drawbacks are overcome by deep learning techniques that perform a seamless feature extraction from the input using conventional gradient-based methods or propagation of activation differences if the interpretability of features is essential (Shrikumar, Greenside & Kundaje, 2017). With the common availability of data and an ever-increasing computing power, deep learning approaches, such as convolutional neural networks (CNNs), proved to be very efficient and outperformed traditional approaches. Through their successive use of convolutional filters, pooling operations, and fully-connected layers, they mimic how the brain works as they lead the resulting network to focus on features that are crucial in solving supervised tasks. Thanks to their potential to leverage the information contained in considerable amounts of input data, they appear as a way to automatically construct features that we would have had to handcraft ourselves otherwise.

The popularity of deep learning applications in the field of computational biology, bioinformatics, and medical informatics has drastically increased the last years (Angermueller et al., 2016; Jones et al., 2017; Min, Lee & Yoon, 2017). While deep networks, such as sparse auto-encoders, recurrent neural networks (RNN) and long short term memory (LSTM) cells have been exploited for protein structure prediction (Lyons et al., 2014; Heffernan et al., 2015; Spencer, Eickholt & Cheng, 2015; Nguyen, Shang & Xu, 2014; Baldi et al., 2000; Sonderby & Winther, 2014; Di Lena, Nagata & Baldi, 2012) and protein classification (Asgari & Mofrad, 2015; Hochreiter, Heusel & Obermayer, 2007; Sonderby et al., 2015), convolutional architectures are not very common and have been used mainly for gene expression regulation (Alipanahi et al., 2015; Lanchantin et al., 2016; Zeng et al., 2016; Kelley, Snoek & Rinn, 2015; Zhou & Troyanskaya, 2015) and DNA/RNA binding (Jones et al., 2017). CNNs have been applied for the prediction of protein properties by Lin, Lanchantin & Qi (2016) and in combination with RNNs comprised of LSTM cells by Li, Zhang & Liu (2017) for enzyme function prediction based on sequence information. The closest to our work is a 2D CNN ensemble proposed by Zacharaki (2017) for EC which exploits mainly, but not purely, the protein structure. It achieved 90.1% accuracy on a benchmark of 44,661 enzymes from the PDB database. The protein structure was represented by multiple 2D feature maps characterizing the backbone conformation (torsion angles) and the (pairwise) amino acids distances. The results showed that the purely structural features (torsion angles) had limited contribution. This may be related to their global nature coming from the 2D representation, which fails to characterize the local 3D shape.

Recently, architectures directly dealing with 3D structures were tested on various datasets. Maturana & Scherer (2015) extended the use of CNNs from images to volumes with a 3D CNN approach called VoxNet. They showed that this strategy could be effective in solving tasks that lie in the 3D space by benchmarking it on traditional datasets (from domains such as LiDAR, RGBD, CAD) and subsequently outperforming traditional approaches. The 3D CNN uses as input volumetric data (of size 32 × 32 × 32) containing occupancy information and targets the problem of supervised classification. Other works such as the one of Hegde & Zadeh (2016) also take advantage of a 3D representation of the data towards classification. In their work, the authors present two different representations of it: one via voxels, and the other one via projected 2D pixel images. Their ensemble of networks slightly outperforms VoxNet on the ModelNet10 dataset and comes at a higher computational cost.

## Materials and Methods

### Dataset

The dataset used in this study has been retrieved from the RCSB PDB (http://www.rcsb.org), which contained 63,558 enzymes as of mid-March of 2017. It has been randomly split in training and testing set with the proportions 80/20%. Also, 20% of the training set has been put aside for validation and was later used for model selection. The details of each of those sets have been summed up in Table 1.

**Table 1:** Structure of the dataset.

EC1	EC2	EC3	EC4	EC5	EC6	Total	
7,096	12,081	15,290	2,875	1,703	1,632	40,677	Training
1,775	2,935	3,809	743	488	419	10,169	Validation
2,323	3,717	4,762	858	571	481	12,712	Testing
11,194	18,733	23,861	4,476	2,762	2,532	63,558	Total

### Representation space and occupancy grid

It has been established (Illergard, Ardell & Elofsson, 2009) that structure is far more conserved than sequence in nature. Since evolutionary relationships have been recognized as a confounding factor for EC, choosing an approach that focuses on spatial representation will be particularly robust in that respect. For this reason, we aim at building a model that seamlessly extracts relevant shape features from raw 3D structures. Accordingly, enzymes are represented as a binary volumetric shape with volume elements (voxels) fitted in a cube *V* of a fixed grid size *l* with respect to the three dimensions. Continuity between the voxels is achieved by nearest neighbor interpolation, such that for (*i*, *j*, *k*) ∈ [[0,*l*−1]]^3^ a voxel of vertices

$$(i + \delta x,\,j + \delta y,\,k + \delta z)\quad |\quad (\delta x,\delta y,\delta z) \in {\{ 0,1\} ^3}$$

takes the value 1 if the backbone of the enzyme passes through the voxel, and 0 otherwise. Instead of extracting features from this 3D structure and analyzing the calculated multi-channel 2D feature maps (as performed in Zacharaki, 2017), we introduce this structure to the CNN, which optimizes its internal parameters (weights and biases of convolutional filters) using the backpropagation algorithm (Rumelhart, Hinton & Williams, 1986). The output of the convolutional filters comprises the feature maps that are selected by the method as best performing for the specific classification task.

To construct this shape representation, some preprocessing steps are necessary. First, protein structure is mapped to a grid of a predefined resolution. The selection of grid resolution determines the level of complexity/scale retained for the enzymatic structures. A full resolution is not preferred due to high data dimensionality and because fine local details are less relevant in characterizing enzymes’ chemical reactions. Thus, in order to avoid to get trapped into local minima, side chains are ignored and enzymes are represented exclusively through their “backbone” atoms that are carbon, nitrogen, and calcium.

Additionally, we note that enzymes do not possess any absolute spatial orientation. Unlike objects such as chairs or boats that appear usually with a specific orientation, proteins can have any orientation in 3D conformational space, thus the Cartesian coordinates defined by the model stored in PDB represent only a frozen in space and time snapshot of an overall highly dynamic structural diversity. The orientation is irrelevant to the properties of the protein. This observation underlines the need of either a rotation invariant representation or of a convention that makes output structures comparable one to another based on the definition of an intrinsic coordinate system. We define as origin of this intrinsic coordinate system the consensus barycenter of the protein as it is defined by taking into account only the four atoms of the backbone for each residue, and as axes the principal directions of each enzyme calculated by principal component analysis. Each structure is rotated around its center and the three principal directions of the enzyme aligned with the three axes of the Cartesian coordinate system. Instead of defining a common reference frame and aligning the objects before building the prediction model, other works (Brock et al., 2016; Hegde & Zadeh, 2016; Maturana & Scherer, 2015) applied rotations around relevant axes for data augmentation. We decided to spatially normalize the data instead of arbitrarily augmenting them (by random rotations) because the number of samples is already big enough and an adequate sampling of all possible orientations would lead to an extremely large dataset difficult to handle. However, similarly to these works the definition of orientation includes uncertainties about the direction (left-right, bottom-up), which we tackled also by data augmentation.

**Figure 1:**
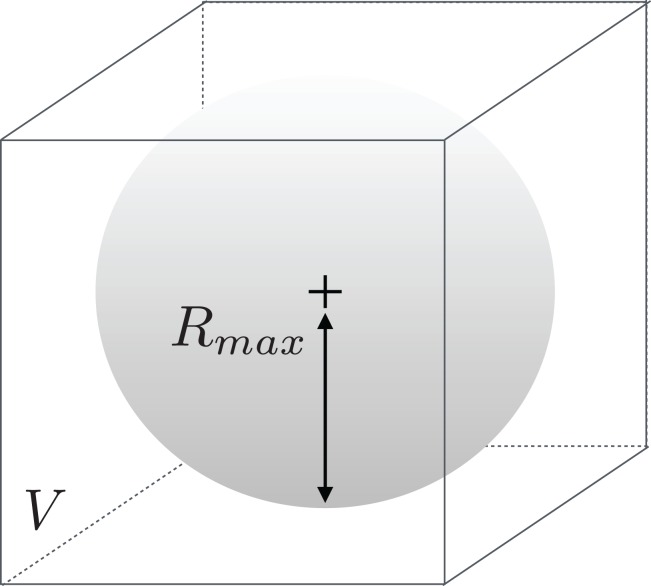
Illustration of the meaning of *R*_max_ with respect to volume *V*.

Another critical part of the process is to determine *how* the enzymes should be fit in their volumes, as those can be of all types of shapes and sizes. Should we scale enzymes separately to make them fit in their respective volumes? Or, on the contrary, should we scale all enzymes in a uniform manner? We choose to select the second option because of two reasons. First, doing otherwise would lead enzymes to be represented at different resolutions. Second, biological considerations invite us to make the convolutional network aware of the size difference between samples, as those may be an implicit feature regarding class determination. We already know that our source files provide the coordinates of the enzymes at a same scale. After all, proteins are comprised of various combinations of equally sized amino acids. This scaling issue is therefore equivalent to determining a maximum radius *R*_max_ so that the atom occupancy information contained in the sphere centered on the barycenter of the enzyme and of radius *R*_max_ fits into *V*. This situation is illustrated in Fig. 1.

*R*_max_ has to be large enough so that a sufficient number of enzymes fit the most of their volume inside *V*. Conversely, it also has to be small enough so that most enzymes are represented at a satisfactory resolution.

As a result, a homothetic transformation with center *S* and ratio λ defined by

$$S\;{\rm{center}}\;{\rm{of}}\;V\;{\rm{and}}\;\lambda = \left\lfloor {{l \over 2} - 1} \right\rfloor \times {1 \over {{R_{\max }}}}$$ (1)

is performed on all enzymes to scale them to the desired size.

When *l* is low, the grid is coarse enough so that the voxels of the structure have a contiguous shape. Conversely, big volumes tend to separate voxels, which engender “holes.” In that case, consecutive backbone atoms $(\overrightarrow {{A_i}}, \overrightarrow {{A_{i + 1}}})$ are interpolated by *p* regularly spaced new points computed by

$${{(p - k + 1) \times \overrightarrow {{A_i}} + k \times \overrightarrow {{A_{i + 1}}} } \over {p + 1}}$$ (2)

where *k* varies from 1 to *p*. The latter is determined empirically beforehand on enzymes of the training set.

A last preprocessing step is to remove potential outliers from the volume. This is done by eliminating voxels that do not have any immediate neighbor.

**Figure 2:**
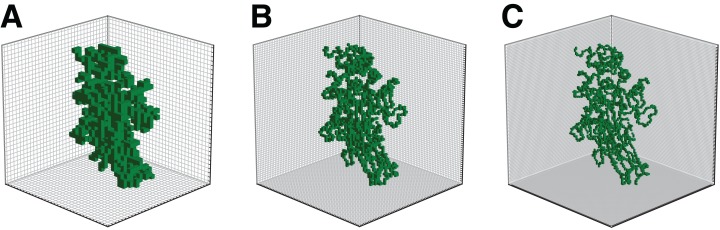
Illustration of enzyme 2Q3Z for grid sizes *l* = 32 (A), *l* = 64 (B), and *l* = 96 (C).

The illustration of the output volume obtained for different grid sizes has been provided in Fig. 2.

### Data augmentation

As noted earlier, differences in spatial orientation had to be considered so that volumes can be comparable one to another.

Keeping the orientation constant, we perform data augmentation by applying transformations that preserve the principal components along the three axes, i.e., flips and combination of flips. The number of possible transformations applicable to each protein is 2^3^−1.

During training, all samples go through the process described in Algorithm 1.

**Algorithm 1:** Summary of the preprocessing steps done to each enzyme at training time

**Data**: *N* training enzymes, grid size of *l*, homothetic transformation ratio λ, *p* interpolations, probability *p*_flip_ to flip with respect to each axis.	
**Input**: Raw coordinates contained in PDB files	
**Output**: Volumes of binary voxels representing backbone atoms occupancy	
1	**foreach** *of the N enzymes of the training set* **do**
2	Step 1: structural information extraction
3	Extract coordinates of backbone atoms from its PDB file
4	Step 2: holes completion
5	Interpolate consecutive backbone atoms by *p* new points
6	Step 3: size adjustment
7	Center barycenter *S* of the coordinates on (0, 0, 0)
8	Homothetic transformation of each point with center *S* and ratio λ
9	Step 4: enzyme orientation
10	Principal component analysis (PCA) transformation
11	Step 5: random augmentation
12	**if** *True with probability p*_flip_ **then**
13	Flip coordinates with respect to the origin along *x*—*axis*
14	**if** *True with probability p*_flip_ **then**
15	Flip coordinates with respect to the origin along *y*—*axis*
16	**if** *True with probability* *p*_flip_ **then**
17	Flip coordinates with respect to the origin along *z*—*axis*
18	Step 6: voxelization
19	Center barycenter *S* of the coordinates on $\left( {{l \over 2},{l \over 2},{l \over 2}} \right)$
20	Transform coordinate points into binary voxels

### Architecture

**Figure 3:**
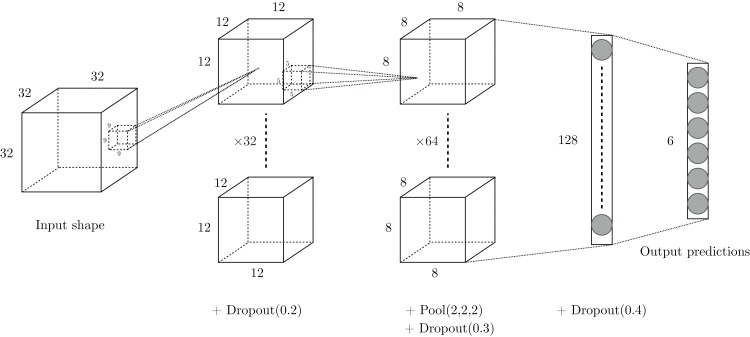
Drawing of the architecture selected for our experiments.

We considered shallow architectures in order to develop a framework that can be trained with common computational means. A grid search of configurations has been conducted on the training set and led us to consider the two-layer architecture presented in Fig. 3: input volumes of size 32 × 32 × 32, containing the structural coordinates of the enzymes encoded in voxels, first go through a convolutional layer of 32 filters of size 9 × 9 × 9 with stride 2. Then, a second convolutional layer of 64 filters of size 5 × 5 × 5 with stride 1 is used, followed by a max-pooling layer of size 2 × 2 × 2 with stride 2. Finally, there are two fully-connected layers of 128 and six (the number of classes) hidden units respectively, concluded by a softmax layer that outputs class probabilities corresponding to the six first-level EC numbers.

Several components of the VoxNet architecture depicted by Maturana & Scherer (2015) have also been investigated in our network. Leaky ReLU with parameter α = 0.1 is used as activation layer after each convolutional layer. The L2 regularization technique of strength λ = 0.001 is applied on the network’s layers. Overfitting is also tackled using dropout throughout the network.

In summary, this model contains 804,614 distinct parameters (including biases), which is approximately 13% less than for the VoxNet architecture.

Additionally, the Adam optimizer introduced by Kingma & Ba (2015) has been chosen for its computational efficiency and low need of hyper-parameter tuning.

The categorical cross entropy is used as loss function, since the latter can be adapted to multiclass classification. Two approaches are considered for computing this loss. The first one assesses misclassification error irrespectively of individual class sizes, whereas the second one takes class imbalance into account and uses customized penalization weights for each class. In this second approach, the aim is to compensate the lack of training samples in under-represented classes by providing a greater penalization to the loss in the event of misclassification than for larger classes.

The cross entropy loss is given by

$${\cal L} = - \sum\limits_{x \in {\rm{set}}} {\sum\limits_{i{\kern 1pt} = {\kern 1pt} 1}^6 {{w_i}} } \cdot{{\rm{\delta }}_{x,i}}\cdot\log ({\hat p_{x,i}})$$ (3)

where ${\hat p_{x,i}}$ is the predicted probability of enzyme *x* belonging to class EC*i*, δ_*x*,*i*_ the quantity equal to 1 only if enzyme *x* belongs to class EC*i*, and *w_i_* the weight associated to class EC*i*. For the first approach, all weights *w_i_* are taken equal to 1. For the weighted approach, we chose weights according to the formula

${w_i} = \displaystyle{{\mathop {\max }\limits_{j \in [[1;6]]} \# ({\rm{EC}}j\;{\rm{training}}\;{\rm{enzymes}})} \over {\# ({\rm{EC}}i\;{\rm{training}}\;{\rm{enzymes}})}}$ (4)

which increases the contribution of the under-represented classes by an amount inversely proportional to their size and in respect to the largest class.

### Metrics

Among the various multi-class metrics that have been studied by Sokolova & Lapalme (2009), we selected the most representative ones to assess the model’s performance. The metrics are based on the confusion matrix whose elements *C*(*i*, *j*) with *i*, *j* ∈ [[1, 6]] indicate the number of enzymes that belong to class EC*i* and are predicted as belonging to class EC*j*. They include:

- *Accuracy* which captures the average per-class effectiveness of the classifier:
  $${\rm{Accuracy}} = {{\sum\limits_{i = 1}^6 C (i,i)} \over {\sum\limits_{i,j = 1}^6 C (i,j)}}$$
- *Precision, recall* and *F*1 score which are calculated per class EC*i*:
  $${\rm{Precisio}}{{\rm{n}}_{{\rm{EC}}i}} = {{C(i,i)} \over {\sum\limits_{j = 1}^6 C (j,i)}}\;\;\;\;\;\;\;\;\;\;\;\;\;\;\;\;\;\;\;\;{\rm{Recal}}{{\rm{l}}_{{\rm{EC}}i}} = {{C(i,i)} \over {\sum\limits_{j = 1}^6 C (i,j)}}$$
  $$F{1_{{\rm{EC}}i}} = 2 \times {{{\rm{Precisio}}{{\rm{n}}_{{\rm{EC}}i}} \times {\rm{Recal}}{{\rm{l}}_{{\rm{EC}}i}}} \over {{\rm{Precisio}}{{\rm{n}}_{{\rm{EC}}i}} + {\rm{Recal}}{{\rm{l}}_{{\rm{EC}}i}}}}$$

For each class, precision gives us an idea of the proportion of correctly classified enzymes among enzymes that have been classified in that class, while recall highlights the proportion of correctly classified enzymes among enzymes that actually belong to that class.

- *Macro precision, recall* and *F*1 score which express average performance over the six enzyme classes:
  $${\rm{Precisio}}{{\rm{n}}_M} = {1 \over 6}\sum\limits_{i = 1}^6 {{\rm{Precisio}}{{\rm{n}}_{{\rm{EC}}i}}} \;\;\;\;\;\;\;\;\;\;{\rm{Recal}}{{\rm{l}}_M} = {1 \over 6}\sum\limits_{i = 1}^6 {{\rm{Recal}}{{\rm{l}}_{{\rm{EC}}i}}} $$
  $$F{1_M} = 2 \times {{{\rm{Precisio}}{{\rm{n}}_M} \times {\rm{Recal}}{{\rm{l}}_M}} \over {{\rm{Precisio}}{{\rm{n}}_M} + {\rm{Recal}}{{\rm{l}}_M}}}$$

### Final decision rule

At testing time, several approaches are considered for determining an enzyme’s final class.

- The “None” strategy consists of making a prediction based on the model of the 3D shape without any transformation.
- In the “flips” approach, the 2^3^−1 possible combinations of flipped volumes are generated and introduced to the classifier. The final class prediction $\hat i$ is either:
  – the class of maximum total probability (*probability*-based decision), determined by
    $$\hat i = \arg \;\mathop {\max }\limits_{i \in [[1,6]]} \sum\limits_{j = 1}^n {{{\hat p}_{i,j}}} $$
    with *n* the total number of flips used during inference and ${\hat p_{i,j}}$ the probability to belong to class *i* during flip *j*
  – or the class selected by majority voting (*class*-based decision), computed through the formula
    $$\hat i = \arg \;\mathop {\max }\limits_{i \in [[1,6]]} \sum\limits_{j = 1}^n {{{{{\hat \delta }}}_{i,j}}} $$
    where ${{{{{\hat \delta }}}_{i,j}}}$ equals 1 if at flip *j*, the enzyme is predicted to belong to class *i*, 0 otherwise.
- The “weighted flips” (W. flips) strategy is also based on fusion of decisions produced for each flipped volume, but this one weights each decision by a different coefficient, such as ${1 \over {{{\rm{\delta }}_x} + {{\rm{\delta }}_y} + {{\rm{\delta }}_z} + 1}}$, which highlights transformations with the least number of flips. Just like the previous approach, the final result can be determined from either a probability- or class-based viewpoint.

### Hyperparameter selection

#### Radius R_max_ and interpolation parameter p

**Figure 4:**
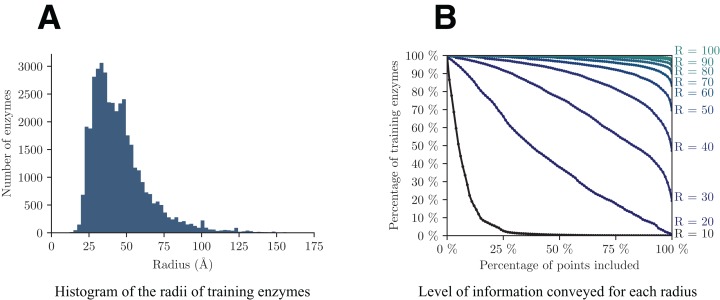
Analysis of the distribution of the radii among training enzymes (A) and corresponding level of information conveyed (B).

A crucial point in the presented approach is to make a cogent selection of *R*_max_. As previously discussed, this parameter controls the trade-off between the level of information (*l*) retained in each volume and the resolution with which they are conveyed (*R*_max_). Figure 4 shows the analysis of the dataset from these two perspectives. The graph on the left helps us assess the minimum radius for which a decent amount of enzymes will be totally included in the volume, whereas the graph on the right highlights the quantity of information retained by each radius.

In details, from Fig. 4A we can see that the majority of enzymes can fit in a sphere with radius between 25 and 75 Å, with a peak around 35 Å. In Fig. 4B, each point (*x*, *y*) on a curve of radius *R* shows the percentage *y* of training enzymes that have at least *x* points included within radius *R* around their respective enzyme barycenter. Based on both graphs, we select a value of *R*_max_ = 40, that is big enough to capture more than half of enzymes in *V*, but small enough so that the smallest enzymes have radii of at least half of *R*_max_.

Empirical observations show that a grid of *l* = 32 does not require any atom interpolation. Therefore, *p* is set to zero for our computations. For denser grids, e.g., with grid size *l* = 64 or 96, appropriate *p* values were *p* = 5 and *p* = 9, respectively.

#### Random selection of transformations and samples

Regarding data augmentation, the probability of enzyme flip along each axis has been set to 2/10. That way for each enzyme, higher numbers of flips have a lower probability of happening. Also for each pass, approximately half randomly selected enzymes are to be augmented by flips (or a combination of flips). This process is useful, as it will help us obtaining a robust classifier.

## Results

**Figure 5:**
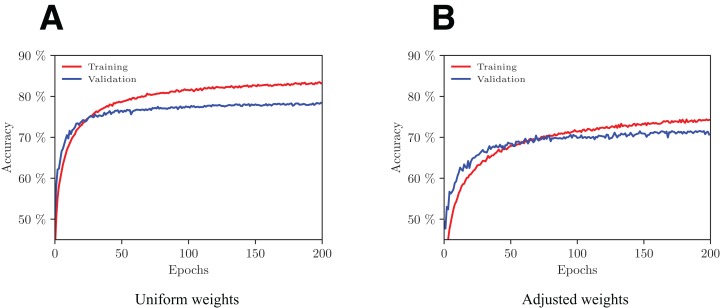
Evolution of the network’s performance during the training process with uniform (A) and adjusted (B) weights.

We trained the model with and without weights adjustment in the loss function using a fivefold cross-validation scheme. The evolution of performance on one of the folds with increasing number of epochs is shown in Fig. 5. With the adopted configuration of hyperparameters, the model converges after 200 epochs.

Cross-validation results associated with our experiments are shown in Table 2. For each fold, both networks with and without class weights (respectively called “adapted” and “uniform” models) have been trained on a training set of 50,846 samples and tested on the remaining 12,712 samples. The cross-validation performance of each model has been evaluated using each of the five inference schemes described earlier and with both micro- and macro-level metrics.

**Table 2:** Fivefold cross-validation results.

Weights			Uniform					Adapted				
Decision			None	Probabilities		Classes		None	Probabilities		Classes	
Augmentation				Flips	W. flips	Flips	W. flips		Flips	W. flips	Flips	W. flips
Accuracy			**0.776** ± 0.002	0.752 ± 0.004	0.771 ± 0.002	0.748 ± 0.002	0.765 ± 0.002	0.711 ± 0.006	0.719 ± 0.007	0.733 ± 0.004	0.710 ± 0.007	0.722 ± 0.008
Precision	EC	1	0.841 ± 0.019	0.900 ± 0.021	**0.905** ± 0.022	0.859 ± 0.026	0.884 ± 0.022	0.752 ± 0.026	0.786 ± 0.036	0.796 ± 0.035	0.717 ± 0.036	0.769 ± 0.029
		2	0.761 ± 0.021	0.753 ± 0.040	0.768 ± 0.035	0.725 ± 0.035	0.758 ± 0.032	0.784 ± 0.015	0.784 ± 0.024	**0.799** ± 0.026	0.760 ± 0.026	0.789 ± 0.025
		3	0.740 ± 0.017	0.686 ± 0.026	0.706 ± 0.023	0.702 ± 0.025	0.706 ± 0.022	**0.774** ± 0.020	0.735 ± 0.030	0.750 ± 0.030	0.743 ± 0.030	0.752 ± 0.028
		4	0.927 ± 0.018	**0.972** ± 0.008	**0.972** ± 0.011	0.970 ± 0.012	0.964 ± 0.007	0.696 ± 0.032	0.836 ± 0.042	0.833 ± 0.042	0.835 ± 0.036	0.792 ± 0.037
		5	0.888 ± 0.013	0.967 ± 0.012	**0.973** ± 0.010	0.971 ± 0.015	0.954 ± 0.010	0.462 ± 0.046	0.584 ± 0.070	0.589 ± 0.060	0.591 ± 0.074	0.550 ± 0.067
		6	0.849 ± 0.037	**0.977** ± 0.029	0.971 ± 0.031	**0.977** ± 0.024	0.953 ± 0.038	0.324 ± 0.040	0.330 ± 0.052	0.347 ± 0.049	0.351 ± 0.049	0.339 ± 0.051
	Macro		0.834 ± 0.011	0.876 ± 0.007	**0.883** ± 0.009	0.867 ± 0.007	0.870 ± 0.009	0.632 ± 0.006	0.676 ± 0.008	0.685 ± 0.006	0.666 ± 0.010	0.665 ± 0.009
Recall	EC	1	**0.747** ± 0.013	0.686 ± 0.021	0.715 ± 0.018	0.706 ± 0.018	0.718 ± 0.018	0.746 ± 0.008	0.722 ± 0.018	0.745 ± 0.012	0.735 ± 0.014	0.739 ± 0.009
		2	0.784 ± 0.021	0.766 ± 0.033	**0.785** ± 0.028	0.783 ± 0.028	0.777 ± 0.026	0.657 ± 0.026	0.646 ± 0.040	0.665 ± 0.034	0.650 ± 0.035	0.656 ± 0.032
		3	0.880 ± 0.015	0.907 ± 0.021	**0.909** ± 0.019	0.881 ± 0.023	0.900 ± 0.019	0.753 ± 0.018	0.790 ± 0.031	0.800 ± 0.029	0.766 ± 0.032	0.781 ± 0.028
		4	0.645 ± 0.008	0.541 ± 0.006	0.583 ± 0.009	0.514 ± 0.007	0.583 ± 0.008	**0.717** ± 0.019	0.698 ± 0.019	0.711 ± 0.016	0.678 ± 0.015	0.708 ± 0.021
		5	0.525 ± 0.016	0.416 ± 0.017	0.462 ± 0.012	0.402 ± 0.017	0.458 ± 0.009	0.663 ± 0.019	0.663 ± 0.021	**0.667** ± 0.020	0.643 ± 0.015	0.665 ± 0.019
		6	0.382 ± 0.020	0.233 ± 0.022	0.283 ± 0.019	0.203 ± 0.017	0.284 ± 0.018	0.638 ± 0.037	0.664 ± 0.041	**0.665** ± 0.047	0.633 ± 0.036	0.655 ± 0.043
	Macro		0.660 ± 0.003	0.592 ± 0.007	0.623 ± 0.005	0.582 ± 0.007	0.620 ± 0.005	0.695 ± 0.007	0.697 ± 0.009	**0.709** ± 0.008	0.684 ± 0.008	0.701 ± 0.010
F1	EC	1	0.791 ± 0.007	0.778 ± 0.008	**0.799** ± 0.008	0.774 ± 0.006	0.792 ± 0.005	0.749 ± 0.011	0.752 ± 0.011	0.769 ± 0.013	0.725 ± 0.014	0.754 ± 0.010
		2	0.772 ± 0.007	0.758 ± 0.005	**0.775** ± 0.005	0.752 ± 0.007	0.766 ± 0.006	0.712 ± 0.010	0.707 ± 0.016	0.724 ± 0.013	0.700 ± 0.013	0.715 ± 0.013
		3	**0.803** ± 0.005	0.781 ± 0.009	0.795 ± 0.008	0.781 ± 0.007	0.791 ± 0.007	0.763 ± 0.007	0.760 ± 0.006	0.773 ± 0.005	0.753 ± 0.008	0.766 ± 0.007
		4	0.761 ± 0.007	0.695 ± 0.005	0.728 ± 0.007	0.672 ± 0.005	0.726 ± 0.005	0.706 ± 0.011	0.761 ± 0.015	**0.767** ± 0.014	0.748 ± 0.012	0.747 ± 0.016
		5	**0.659** ± 0.015	0.582 ± 0.018	0.625 ± 0.013	0.569 ± 0.019	0.619 ± 0.010	0.543 ± 0.032	0.618 ± 0.037	0.624 ± 0.032	0.613 ± 0.043	0.600 ± 0.043
		6	**0.526** ± 0.021	0.376 ± 0.029	0.438 ± 0.023	0.336 ± 0.023	0.438 ± 0.022	0.428 ± 0.031	0.437 ± 0.042	0.452 ± 0.034	0.449 ± 0.036	0.444 ± 0.041
	Macro		**0.737** ± 0.003	0.706 ± 0.004	0.730 ± 0.003	0.696 ± 0.003	0.724 ± 0.002	0.662 ± 0.006	0.687 ± 0.007	0.697 ± 0.004	0.675 ± 0.007	0.683 ± 0.008

**Note:**

Best performance for each indicator is shown in bold.

Overall, the model that performs best in terms of both accuracy (77.6%) and macro *F*1 (73.7%) is the one with uniform weights using no data augmentation.

Precision per class on under-represented classes (EC4, 5, 6) is far better on uniformly weighted models compared to adapted models, with best scores being 97.2%, 97.3% and 97.7%, versus 83.6%, 59.1% and 35.1% respectively. Over-represented classes (EC2, 3) have roughly the same performance in those two types of model.

It is worth noting that by augmenting the data, the precision per class is noticeably improved on under-represented classes (EC4, 5, 6) in both uniform and adapted models. In fact, in the uniformly weighted framework, the best data augmented models achieve 97.2%, 97.3%, and 97.7% versus 92.7%, 88.8%, and 84.9% for the non-augmented one for EC4, 5, 6 respectively. An interpretation to this fact is that the configurations produced with flips are good at imitating plausible configurations that hold similar EC properties. Similarly, using data augmentation during inference for the adapted framework achieves better performance than when it is not used, with 83.6%, 59.1%, and 35.1% versus 69.6%, 46.2%, and 32.4% for EC4, 5, 6 respectively.

Recall per class on over-represented classes (EC2, 3) are best with uniformly weighted models (78.5% and 90.9% respectively). It is worth noting that the adapted model outperforms the uniform one on under-represented classes (EC4, 5, 6) with 71.7%, 66.7%, 66.5% recall per class respectively, compared to 64.5%, 52.5%, and 38.2% respectively on uniform models. This drastic performance increase was once again expected, as we know that the adapted-weights network was heavily penalized for its errors on small classes during the training process.

Uniformly weighted models perform best in terms of *F*1 scores, ranging from 52.6% for the smallest class (EC6) to 79.9% for EC1, with a macro *F*1 of 73.7%.

### Computation time

Our architecture was implemented on Python 3 using Keras (Chollet, 2015) on top of a GPU-enabled version of TensorFlow (Abadi et al., 2015). Enzyme information has been extracted using the open-source module BioPython, a fast and easy-to-use tool presented by Cock et al. (2009). The complete training of our model on 50,846 samples took about 5 h on an Intel i7 6700K machine with 32 GB of RAM and a GTX 1080 graphics card. During the training phase, the code computes batches in parallel and stores them in a queue in real-time, so that the main computational burden comes from neural network calculations and not the representation process. The average prediction time of the function of a single enzyme was about 6 ms without flips, or 50 ms with flips.

## Discussion

The general trend is that uniformly weighted models perform well in terms of macro accuracy, macro precision as well as macro *F*1 while adapted models are slightly better in macro recall. More particularly, on under-represented classes (EC4, 5, 6), models perform better in terms of precision per class when using flip data augmentation, which means that using data augmentation increases reliability on predictions. This can be explained by the fact that the classifier has more examples at hand, which makes its predictions more robust. Interestingly, on under-represented classes, uniform models have the highest precision per class and the lowest recall per class while adapted models are exactly the opposite: they have the lowest precision per class and the highest recall per class. The interpretation of this clear trend is that enzymes coming from under-represented classes are not always recognized by uniform classifiers as they are biased towards enzymes from over-represented class because the classifier does not correct for class imbalance. On the contrary, enzymes from under-represented classes are well recognized by adapted classifiers as they account for class imbalance, but this comes at a price: by predicting more enzymes as being in those under-represented classes (false positives), the classifier tends to make a lot more mistakes which leads to a low precision per class.

### Further improvements of the method

In parallel, we investigated possible improvements in the architecture. We introduced batch normalization (Ioffe & Szegedy, 2015) at several positions of the network and repeated the same experiments as before. Those were placed after every activation layer, as suggested by Mishkin, Sergievskiy & Matas (2017). Also, Leaky ReLU activation layers were replaced by PReLU (He et al., 2015) layers which enable the network to learn adaptively the Leaky ReLU’s best α parameter. Although these changes led the network to converge faster to a stable optimum, the final performance increase was of an order of magnitude of only one percent and came at a higher computational cost.

**Figure 6:**
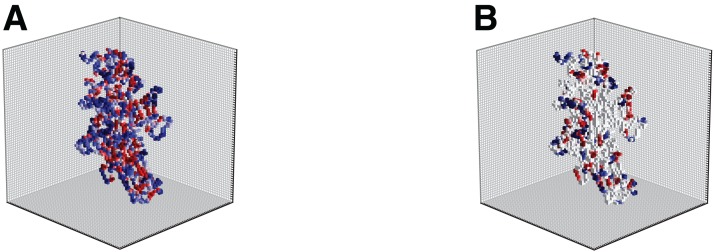
Illustration of the hydropathy (A) and charge (B) attributes incorporated into the shape model for enzyme 2Q3Z. Each attribute is illustrated in color for better visualization, but it actually corresponds to a single channel.

In the following, we considered the transition from a binary representation capturing only shape information to “gray-level” representation capturing also information content. Several biological indicators such as the hydropathy index (Kyte & Doolittle, 1982), or isoelectric points (Wade, 2002) can be used to better describe local properties of amino acids that are the building blocks of the protein structure. From the perspective of computational analysis, these attributes can be incorporated into the representation model by attaching to the shape also appearance information. Figure 6 illustrates this idea showing the volumetric representation of two different attributes (hydropathy on the left and charge on the right). The different attributes, if up to three, can also be merged into a single structure visualized in color (RGB) scale. Different approaches to handle multi-channel volumetric data (images) by CNNs have recently been presented in works such as those of Zhang et al. (2017), Cao et al. (2016), Kamnitsas et al. (2017) and Nie et al. (2016). The literature however on the use of deep networks for 3D shapes with multi-channel appearance is limited. As preliminary analysis, we applied EnzyNet without modification on the architecture, but with re-training for the adjustments of the weights of the convolutional kernels. We introduced to the network either a single attribute (as a binary 3D image or gray-level 3D image), such as shape, hydropathy and isoelectric points, or a combination of these different attributes. Two other ways of information combination were examined: either each attribute was introduced as a different channel in the CNN, or the outputs of the single channel networks were combined through a fusion rule. However, all methods showed an increase of the order of magnitude of one percent. Further investigation will be required to appropriately harness this added information.

Furthermore, in the current study a relatively small grid size was selected due to computational limitations. However, as can be seen in Fig. 2 the details of the spatial structure of proteins can be better differentiated for higher values of *l*. The next step of this work would be to adapt the current architecture accordingly into a similar network that processes higher-resolution volumes. This could enable the network to capture more subtle features and potentially boost the performance of the classifier. On the other hand, the model performance using a finer representation might be more sensitive to possible inaccuracies in protein structure prediction. Initial tests showed that the appearance of artifacts in the location of atoms at *l* = 64 or 96 had a negligible impact on accuracy.

Wei et al. (2015) present a method that enables 3D CNN to deal with multi-label classification problems. This approach is interesting for us as it allows the extension of our method to the classification of multi-label enzymes. The obtained performance could subsequently be compared to previous work Amidi et al. (2017).

Other approaches based on the 3D representation of enzymes are also possible. Brock et al. (2016) performed very well on the ModelNet dataset using generative and discriminative modeling. Their voxel-based autoencoder is helpful for assessing the key features that are correctly learned from the 3D shapes. We could elaborate this information in order to identify significant features in a pre-training phase aiming to obtain better prediction performance.

## Additional Information and Declarations

### Data Availability

The following information was supplied regarding data availability: GitHub: https://github.com/shervinea/enzynet.
